# Neurosyphilis with Concomitant Cryptococcal and Tuberculous Meningitis in a Patient with AIDS: Report of a Unique Case

**DOI:** 10.1155/2017/4103858

**Published:** 2017-08-27

**Authors:** Jose Armando Gonzales Zamora, Luis Alberto Espinoza, Rita N. Nwanyanwu

**Affiliations:** Division of Infectious Diseases, Department of Medicine, University of Miami, Miller School of Medicine, Miami, FL 33136, USA

## Abstract

Meningitis in individuals living with acquired immunodeficiency syndrome (AIDS) is most frequently infectious in origin and usually due to opportunistic infections. The most common pathogens are* Cryptococcus neoformans* and* Mycobacterium tuberculosis*.* Treponema pallidum* causes neurosyphilis and can complicate HIV infections at any time after the initial infection. Simultaneous infections of the central nervous system caused by these pathogens are very uncommon even in the setting of severe immunosuppression. We report the case of a newly diagnosed HIV/AIDS young man who was found to have neurosyphilis with* Cryptococcus* meningitis. After a few weeks of treatment and initiation of antiretroviral therapy, he was also diagnosed with tuberculous meningitis, which was probably unmasked by the development of immune reconstitution inflammatory syndrome (IRIS). To the best of our knowledge, this is the only case of reported neurosyphilis and meningitis caused concomitantly by* Cryptococcus* and* Mycobacterium tuberculosis.*

## 1. Introduction

*Cryptococcus neoformans* is the leading cause of meningitis in patients with HIV/AIDS. This organism has a propensity to invade the central nervous system under circumstances of severe immunosuppression. Although its incidence has declined in the era of antiretroviral therapy, this infection remains an important cause of mortality in the developing world [1]. Another infection of particular interest in patients with HIV is tuberculosis, which accounts for an important number of meningitis cases in areas where this infection is endemic [2]. The AIDS pandemic has also brought diseases like syphilis to the center of attention in global health. Several case studies have reported an increase in the occurrence of neurosyphilis, with changes in its clinical profile and with earlier and more severe clinical manifestations [3]. Although, independently, these infections are common in HIV infected patients, their concurrent presentation is extremely rare. We present a case of meningitis caused simultaneously by* Treponema pallidum*,* Cryptococcus,* and* Mycobacterium tuberculosis* in a patient with AIDS. To our knowledge, this is the first case reported in the English-language literature.

## 2. Case Description

**Figure 1:**
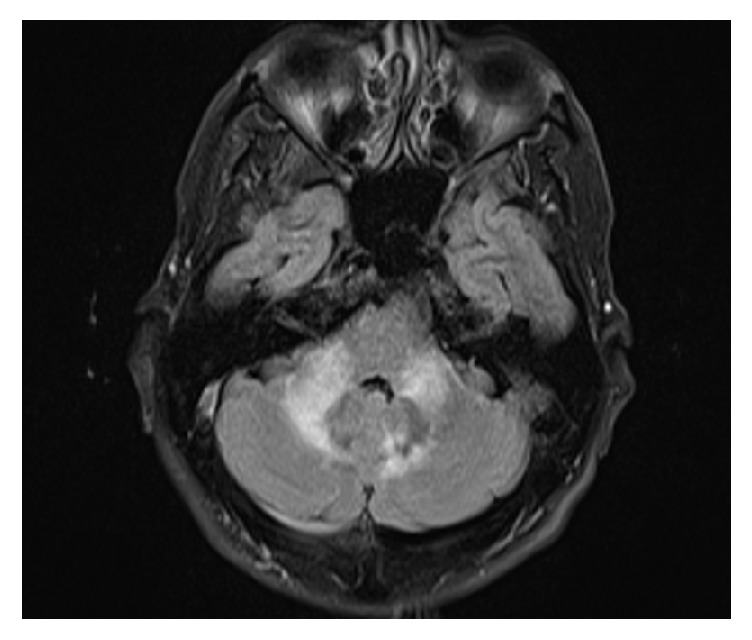
Axial T2-FLAIR MRI showing interval increase of leptomeningeal enhancement and hyperintense signal involving the left middle cerebral peduncle, cerebellar vermis, and bilateral cerebellar hemisphere.

A 43-year-old Hispanic male presented to the hospital with frontal headache, fever, nausea, and vomiting ongoing for 2 weeks. His past medical history was significant for herpes zoster ophthalmicus two years ago. His vital signs on admission were within normal limits. Neurological examination was remarkable for mild cervical stiffness and pain with neck motion. No focal signs were noted. Laboratory studies were remarkable for normal white blood cell count (WBC: 6.7 k/uL), low hemoglobin (10.8 g/dL), and normal platelet count (172 K/uL). Rapid HIV test was reactive. Complete chemistry panel was within normal limits. Chest X-ray did not show any abnormalities. Due to a high suspicion for meningitis, the patient underwent a brain computed tomography (CT) followed by a lumbar puncture. Brain CT scan did not disclose any abnormalities. Cerebrospinal fluid (CSF) analysis revealed high WBC count (73 leukocytes/mm^3^) with lymphocytic predominance (96%), high protein (112 mg/dL), and low glucose (37 mg/dL). The patient was started on ceftriaxone, vancomycin, and acyclovir empirically. Later on, studies revealed a CD4 count of 83 cells/uL and a HIV viral load of 51,080 copies/mL. Serum RPR was reactive with a titer of 1 : 1024 dils. VDRL from CSF was also reactive. Based on these results, the patient was diagnosed with neurosyphilis and intravenous penicillin was started. At this point, ceftriaxone, vancomycin, and acyclovir were discontinued. Over the following three days, the patient reported worsening headache and neck pain accompanied by nausea and vomiting. He also complained of slurred speech and visual hallucinations. Due to lack of response to neurosyphilis treatment, a magnetic resonance imaging was ordered. It showed T2 and flair hyperintense signal involving the left middle cerebral peduncle and vermis and in the right cerebellar hemisphere medial aspect. Lumbar puncture was repeated and CSF analysis showed a higher number of WBCs (267 leukocytes/mm^3^) with lymphocytic predominance (74%), low glucose (8 mg/dL), and high protein (94 mg/dL).* Cryptococcus* antigen from CSF was positive with a titer of 1 : 640 dils. India ink stain and fungal culture were negative. He was started on amphotericin B lipid complex and flucytosine for cryptococcal meningitis. The patient improved clinically over the following week. After two weeks of treatment with amphotericin and flucytosine, he was switched to oral fluconazole 400 mg daily for consolidation therapy. He also completed two weeks of intravenous penicillin for neurosyphilis and was discharged in stable condition with resolution of headaches. After 2 weeks, he started antiretroviral therapy with tenofovir alafenamide/emtricitabine and darunavir-ritonavir. At 1-month follow-up, he complained of malaise and dizziness along with slurred speech and left hand clumsiness. On physical exam, he had an ataxic gait and saccadic eye movements. A repeat MRI showed improvement of leptomeningeal enhancement supratentorially but progression of leptomeningeal enhancement and T2/FLAIR signal abnormality within the posterior fossa (Figure 1). The patient was readmitted to the hospital and underwent a new lumbar puncture. Opening pressure was elevated (26 cm H_2_O). CSF analysis showed 2 WBCs and normal glucose and protein. India ink stain revealed rare encapsulated yeasts.* Cryptococcus* antigen from CSF was 1 : 80 dils. Fungal culture did not show any growth. He was restarted on induction therapy with amphotericin B 1 mg/kg/day and flucytosine for a possible relapse of cryptococcal meningitis. After 2 weeks of treatment, his symptoms improved significantly and he was discharged on oral fluconazole 800 mg daily for consolidation therapy. Two weeks after discharge, CSF culture from recent hospital admission grew* Mycobacterium tuberculosis*. Identification was confirmed by PCR restriction analysis- (PRA-)* hsp65*. Antibiogram showed resistance only to streptomycin (Table 1). The patient was started on antituberculosis therapy with isoniazid, rifabutin, pyrazinamide, ethambutol, and cycloserine. His antiretroviral therapy was modified to avoid drug interactions with antituberculosis regimen. Darunavir-ritonavir was switched to dolutegravir and he continued with tenofovir alafenamide/emtricitabine. Given his recent episode of possible* Cryptococcus* relapse, he continued with a high fluconazole dose (800 mg daily) for a prolonged consolidation therapy.

## 3. Discussion

One of the infections presented by our patient was neurosyphilis, which is of particular concern in HIV infected individuals. The immunodeficiency state induced by HIV may alter the natural course of syphilis by reducing the immunologic response to treponemal infection, which facilitates the progression of syphilis [4]. Several studies have shown that the risk of neurosyphilis in HIV infected patients is significantly higher when CD4 count is <350 cells/uL and RPR is >1 : 32 [3]. The clinical manifestations of neurosyphilis are myriad, but the most commonly reported ones in the setting of HIV are meningitis and meningovascular syndrome. There are also asymptomatic cases in which the diagnosis relies exclusively on cerebrospinal fluid analysis. The most frequent abnormalities in CSF are pleocytosis with lymphocytic predominance, elevated protein, and normal or low glucose [5]. These findings are also seen in other types of infections (fungal, mycobacteria); thus, the diagnosis often relies on CSF serology (VDRL or FTA-ABS). Given the protean manifestations of neurosyphilis, the differential diagnosis is broad and includes infectious and noninfectious conditions that cause meningitis, stroke-like phenomena, focal central nervous lesions, dementia, and myelopathies (Table 2). Of note, the occurrence of meningeal infections in association with neurosyphilis is very rare. In patients with HIV/AIDS, the literature is limited to two reports of concomitant neurosyphilis and cryptococcal meningitis and one report of neurosyphilis associated with tuberculous meningitis [6–8]. In our patient, the diagnosis of neurosyphilis was established early in the course of hospitalization with a positive VDRL from CSF, but the lack of response to intravenous penicillin led to an additional work-up to search for concomitant infections.

Another cause of meningitis commonly encountered in HIV/AIDS and developed by our patient is* Cryptococcus*. This opportunistic infection has a propensity to invade the central nervous system in immunocompromised individuals, especially in HIV infected patients with CD4 counts < 100 cells/uL. The presentation of cryptococcal meningitis is typically subacute and symptoms can begin indolently over a period of one to two weeks. The CSF analysis typically reveals a low WBC count (<50) with mononuclear predominance, a mildly elevated protein, and a low glucose concentration; however up to 25% of patients with culture positive cryptococcal meningitis have a normal CSF profile [9]. A positive* Cryptococcus* antigen in CSF, which has a very high sensitivity (99.3%), usually confirms the diagnosis [10]. Our patient did not have a full evaluation for fungal pathogens on his first lumbar puncture, but due to the progression of neurological symptoms a second lumbar puncture was obtained, which yielded a positive* Cryptococcus* antigen. Culture is considered the gold standard with a sensitivity of 86% in HIV infected patients; however, it showed a negative result in our patient [10]. In terms of treatment, an induction phase with amphotericin and flucytosine is typically given for two weeks. This is followed by a consolidation therapy with fluconazole for 8 weeks. After completion of this second phase, maintenance therapy with low doses of fluconazole should be continued for long-term suppression [10]. Nonadherence to maintenance therapy is one of the main causes of cryptococcal meningitis relapse, which can further complicate the treatment of this infection [11]. Our patient had a second admission with progression of his neurological symptoms, which was attributed to a relapse of cryptococcal meningitis. However, his presentation was not fully consistent with a case of microbiologic relapse, mainly because fungal cultures did not isolate any* Cryptococcus neoformans* [11]. CSF analysis of our patient only revealed yeasts in fungal smear, which were probably nonviable forms of* Cryptococcus* and by themselves were not indicative of active fungal replication especially in a patient receiving an adequate treatment. Our patient's paradoxical deterioration might have been secondary to an immune reconstitution inflammatory syndrome (IRIS), which is described in patients with rapid immunologic recovery from antiretroviral therapy [12]. Our patient clearly had a robust response to ART, with a significant decrease in his HIV viral load from 51080 copies/mL to 488 copies/mL and a marked increase of his CD4 count from 83 cells/uL to 307 cells/uL over the course of two months. In terms of treatment, cryptococcal meningitis IRIS requires evacuatory lumbar punctures and steroids in severe cases; conversely, relapse from cryptococcal meningitis is treated with a full course of antifungal therapy. Some cases cannot always be distinctly classified as either microbiologic relapse or IRIS, and the treatment sometimes has to cover both possibilities. In this regard, some authors have reported cases consistent with cryptococcal meningitis IRIS in which brain biopsy ultimately revealed active replication of fungal organisms [13]. Although our patient did not meet the criteria for relapse, we decided to treat him with antifungals to also cover the possibility of relapse secondary to an infection with low fungal burden undetected by cultures. Interestingly, IRIS not only led to paradoxical worsening in our patient, but also unmasked an occult opportunistic infection such as meningeal tuberculosis.

As evidenced in our patient, the diagnosis of meningeal tuberculosis is very challenging. Typically, the CSF analysis shows elevated protein, low glucose, and mononuclear pleocytosis, findings that are also seen in fungal meningitis [14]. The culture of* Mycobacterium tuberculosis* in CSF has a sensitivity of only 37%, but it can increase to 87% after three serial lumbar punctures owing to a low burden of mycobacteria in CSF samples [15]. In our patient, the diagnosis was made by a positive culture obtained from the third CSF analysis, which illustrates the importance of obtaining a high volume of CSF to achieve a diagnosis of meningeal tuberculosis. The diagnostic yield could have been also increased with the performance of molecular tests such as Xpert PCR; unfortunately, this test was not done in our patient.

Meningitis caused simultaneously by* Cryptococcus* and tuberculosis is extremely rare. To our knowledge, there are only four cases in HIV-1 patients reported in the literature [6, 16, 17]. The biochemical characteristics of CSF are very similar in these two infections; however, some authors have pointed out that CSF in cryptococcal meningitis usually reveals a mild increase in lymphocytes and that finding significant lymphocytosis should raise the concern for a concomitant pathology [6]. Our initial patient CSF analysis did present significant lymphocytosis; however, subsequent CSF parameters showed only a few lymphocytes which did not allow any distinction between cryptococcal and tuberculous meningitis. The diagnosis of tuberculosis in our patient was incidental and probably unmasked by the development of IRIS.

The neuroimaging findings in our patient also raised the suspicion for other noninfectious conditions such as primary or metastatic neoplasms. In HIV/AIDS patients, primary CNS lymphoma (PCNSL) constitutes the most common neurologic neoplasm. It typically manifests as a well-defined focal lesion with high degree enhancement on CT and MRI. Leptomeningeal involvement occurs in up to 40% of patients with PCNSL. On the other hand, primary leptomeningeal lymphoma without synchronous cerebral disease is very rare, making up less than 10% of cases [18]. Another condition of particular interest in this population is Kaposi sarcoma (KS), a vascular malignancy that affects mainly mucocutaneous sites. Cerebral involvement has also been described, although uncommonly. The cerebrum is apparently the most frequently affected brain region; however, KS has been also reported in the cerebellum, pons, meninges, and dura matter [19]. On CT scan, KS lesions are usually homogeneous and hyperdense with minimal mass effect and slight surrounding edema. The appearance on MRI is also homogeneous and demonstrates a hyperintense signal on T1- and on T2-weighted images [19, 20]. It is important to be cognizant of these distinctive radiographic features when approaching HIV patients, because malignancy is the cause of brain lesions in up to 36% of cases [21].

## 4. Conclusions

This is the first reported case of meningitis caused by three different pathogens such as* Treponema pallidum*,* Cryptococcus,* and* Mycobacterium tuberculosis*. Our case illustrates the diverse number of infections that may affect the neurologic system in HIV patients with very low CD4 counts. For many of these pathologies, we have highly sensitive tests that allow an early diagnosis and treatment (e.g., cryptococcal antigen and RPR). However, as evidenced in our case, we still have marked limitations in infections such as tubercular meningitis given the low burden of mycobacteria in CSF specimens. Interestingly, tubercular meningitis was unmasked by IRIS in our patient, which allowed the initiation of adequate treatment with excellent outcomes. This suggests that the development of IRIS might be beneficial in patients with pathologies in which the diagnosis is very challenging, especially in cases of concomitant infections.

## Figures and Tables

**Table 1:** Antibiogram of *Mycobacterium tuberculosis*.

Antibiotic	MIC	Susceptibility
Streptomycin	>32	R
Isoniazid	<0.03	S
Rifampin	<0.12	S
Ethambutol	1	S
Rifabutin	<0.12	S
Ethionamide	<0.3	S
Amikacin	0.25	S
Moxifloxacin	0.25	S
para-Aminosalicylic acid	<0.5	S
Cycloserine	8	S
Capreomycin	1.2	S
Levofloxacin	0.5	S

MIC indicated minimum inhibitory concentration (ug/mL). S: susceptible; R: resistant.

**Table 2:** Differential diagnosis of neurosyphilis.

Manifestations of neurosyphilis	Differential diagnosis
(1) Syphilitic meningitis	Tubercular meningitis
	Fungal meningitis: *Cryptococcus neoformans*, endemic mycosis
	Lyme disease
	Listeria monocytogenes
(2) Meningovascular syphilis	Ischemic stroke
	Embolic stroke
	Vasculitis
	Herpes zoster
(3) Gummata	Toxoplasmosis
	Cryptococcoma
	Tuberculoma
	Primary and metastatic neoplasms
(4) General paresis	Psychiatric conditions: delirium, dementia, mania, psychosis
	depression, personality changes
	HIV dementia
	Viral encephalitis: HSV, CMV, VZV, HHV-6
(5) Tabes dorsalis	Epidural abscess
	Subacute combined degeneration of the spinal cord
	HTLV-1
	HIV-associated vascular myelopathy
	CMV myelopathy
	Herpes zoster myelitis Multiple sclerosis

HIV= human immunodeficiency virus; HSV = herpes simplex virus; CMV = cytomegalovirus; VZV = varicella zoster virus; HHV-6 = human herpes virus 6; HTLV-1 = human T-cell lymphotropic virus type 1.
